# Can simple perioperative measures improve quality of recovery following ambulatory laparoscopic surgery in females? An open prospective randomised cohort study, comparing nutritional preoperative drink and chewing gum during recovery to standard care

**DOI:** 10.1016/j.amsu.2019.05.010

**Published:** 2019-06-08

**Authors:** Emma Öbrink, Johanna Lerström, Christian Hillström, Eva Oddby, Jan G. Jakobsson

**Affiliations:** Department for Anaesthesia & Intensive Care, Institution for Clinical Sciences, Karolinska Institutet at Danderyd University Hospital, Stockholm, Sweden

**Keywords:** Day case surgery, Cholecystectomy, General anaesthesia, Quality of recovery, PONV/PDNV

## Abstract

One major goal in modern perioperative anaesthesia care is to facilitate a rapid, yet safe recovery process, with focus on improving time to regained consciousness and subsequent resuming of activities of daily living. Laparoscopic cholecystectomy and gynaecological laparoscopy are a “high volume” procedure commonly performed in young females expecting rapid resumption of health.

The aim of this study was to assess whether it was possible to improve patients’ self-assessed quality of recovery in female patient undergoing laparoscopic cholecystectomy by simple perioperative measures in the form of a preoperative 200 ml nutritional drink and chewing gum during early recovery.

**Methods:**

Patients were randomised to an active group receiving the intervention, and controls provided with standard care only. Patients were followed by questionnaire interviews preoperatively and at 2, 24 and 48 h after surgery. The Quality of Recovery scale (QoR) 15 items and 5 additional questions around gastro-intestinal symptoms were self-assessed by patients at each occasion.

**Result:**

Seventy-three ASA 1–2 female patients’ undergoing elective laparoscopic surgery were included, surgery and anaesthesia was uneventful. The QoR score was significantly higher both at 24 and 48 h, 113 SD 20 vs 101 SD 25 (p = 0.026) and 123 SD 13 vs 111 SD 13 (p = 0.006) in the active group of patients as compared to controls.

**Conclusion:**

Simply providing 200 ml nutritional preoperative drink and chewing gum during recovery was found effective, improving patients assessed quality of recovery.

## Highlights

• One major goal in modern perioperative anaesthesia care is to facilitate a rapid, yet safe recovery process.
• Simply providing 200 ml nutritional preoperative drink and chewing gum during recovery was found effective, improving patients self-assessed quality of recovery.
• Quality of recovery, assessed by a multi dimensional tool (QoR15), in females after laparoscopic surgery is still not complete 48 h after surgery.

## 1 Introduction

Shortening hospital stay after surgery is increasingly implemented, with day, ambulatory and short stay surgery being applied more and more. Quick recovery after surgery is therefore of utterly importance. Much efforts have been put on studying recovery while in hospital; emergence, pain, postoperative nausea and vomiting (PONV) and time to discharge. Pain, nausea and general fatigue are not uncommonly compromising the recovery process especially in female patients after abdominal procedures [1]. Young non-smoking female with a history of PONV needing abdominal surgery and postoperative morphine is at highest risk for PONV/PDNV [2]. Female are in general reporting more symptoms compromising quality of recovery as compared to males [3]. Studies addressing quality of recovery not only while in hospital but following discharge are however warranted.

Maintaining homeostasis and avoid dehydration and depleted energy stores is of importance for overall recovery. The Enhanced Recovery after Surgery (ERAS) concept emphasising the importance of avoiding prolonged fasting has been shown to not only shorten hospital stay but reduce morbidity [4]. Several studies support the concept of carbohydrate loading prior to surgery, improving the recovery, the role of preoperative nutritional drink is however not obvious and the Cochrane systematic review from 2014 could not verify any obvious benefit [5]. Likewise simply letting patient have a xylitol chewing gum has been suggested to facilitate the regain of bowel function, thus counteracting emesis [[6], [7], [8], [9]]. The combine use of carbohydrate preoperative drink and chewing gum during recovery has not, to our knowledge, been priory tested.

Quality of recovery should include not solely pain and PONV/PDNV. Quality of recovery should be assessed broadly, also following discharge and by the patient – self-assessment [10,11]. There are several questionnaires developed and put into use, focusing specifically on assessing quality of recovery (QoR) from the patient's perspective; questions related to patient assessed comfort, emotions such as worry or sadness, need of support from healthcare personnel, level of patient physical independence and experiences of pain [12,13]. One of these questionnaire is the Quality of Recovery-15 (QoR-15), a questionnaire encompassing 15 specific questions on QoR, stratified from the more extensive 40-item Quality of Recovery-40 questionnaire (QoR-40). The QoR-40 is the most extensively used quality-of-recovery-questionnaire providing a global score and sub-scores assessing five dimensions of recovery: patient support, comfort, emotions, physical independence, and pain. The QoR-15 was developed by Stark et al., in 2013, based on the QoR-40, and has been found a valid evaluation of patient assessed QoR when compared with a global QoR Visual Analogue Scale (VAS) [13]. The QoR-15 questionnaire assesses 15 different items on post-operative QoR, asking patients to grade their experiences on an 11-point graded scale, ranging from 0 to 10. Maximum point is thus 150 points. The first questionnaire is filled in by the patient prior to surgery and can therefore be used as baseline compare to the rating postoperatively, and hereby can each subject be its one control.

The aim of the present study was to evaluate if a combination of carbohydrate- and energy-loaded pre-operative drink 2–3 h prior to induction of anaesthesia, combined with xylitol chewing gum during recovery, could improve general quality of recovery.

## 2 Material and methods

The study was approved by the Ethics Committee at the Karolinska Institute (Dnr: 2016/2107–31/4). Patients were informed both verbally and in writing. Written consent form was obtained prior to randomization. The study has been reported in line with the STROCSS criteria [14].

Female patients aged 18–75 years ASA 1–2, scheduled for ambulatory elective laparoscopic cholecystectomy or elective gynaecologic laparoscopy in general anaesthesia between January and December 2017 at Danderyd University Hospital were asked to take part in the study. A total of 98 women were eligible for participation in the study, 11 opted not to participate, 10 could not participate due to logistics and three had language difficulties, in the end 74 were included.

Females classified with ASA 3 or higher, with BMI> 32, liver and/or renal disease, were excluded from the study.

Patients were provided all standard care, and anaesthesia was based on the standard routine of the department. All but five were anaesthetised with total intravenous anaesthesia with remifentanil and propofol and orally intubated after receiving neuromuscular blockage with rocuronium. A modified Apfel score were used to predict the risk of PONV. Since all participants were female they all started up with a risk score of 1. The other items in the modified risk score were smoking-/nicotine use habit, age and history of PONV and/or motion sickness. All the participating women were provided opiate during anaesthesia.

PONV prophylaxis were given according to the attending anaesthetist, all patients were provided with betamethasone further prophylaxis is presented in table perioperative observations.

Patients were randomised in two groups using closed opaque envelope technique.

Females in the active group (n = 37) followed routine pre-operative fasting and postoperative fluid and food regime, received standard care in line with local guidelines at Danderyd University Hospital, and received a two-parted intervention with a carbohydrate and energy loaded drink ProvideXtra^®^ Fresenius Kabi 2–3 h before induction of anaesthesia, and a xylitol chewing gum given post-anaesthesia.

Females in the control group (n = 36) followed routine pre-operative fasting and postoperative fluid and food regime and received standard care according to local guidelines at Danderyd University Hospital.

### 2.1 Self-assessment of quality of recovery

All patients were requested to fill in the QoR15 questionnaire at 4 occasions, preoperative in the holding area, at about 2 h postoperatively when leaving the recovery room, at 24 and 48 h after surgery. We used QoR15 in a Swedish version, which recently has been validated [15]. The 15 basic items in the QoR15 consists of short statements and the patients should respond assess how well the statement adheres on a numeric scale, e.g. item 2: **Been able to enjoy food**? *None of the time* 0 1 2 3 4 5 6 7 8 9 10 All *of the time*. The higher the score the better quality and the best quality experience would end up in a score of 150.(see Table 1).

**Table 1:** QoR 15. (Quality of recovery).

Part A	
How have you been feeling during the last 24 h?	Score between 0 and 10 where 0 is none of the time and 10 is all of the time
Able to breathe easily	
Been able to enjoy food	
Feeling rested	
Have had a good sleep	
Able to look after personal toilet and hygiene unaided	
Able to communicate with family and friends	
Getting support from hospital doctors and nurses	
Ablte to return to work or usual home activities	
Feeling comfortable and in control	
Having a feeling of general well-being	
**Part B**	
Have you had any of the following during the last 24 h?	Score between 0 and 10 where 0 is all of the time and 10 is none of the time.
Moderate pain	
Severe pain	
Nausea or vomiting	
Feeling worried or anxious	
Feeling sad or depressed	

Table 3. QoR-15. The individual scores are added and a total score ranging from 0 to 150 is produced. The score correlates with degree of postoperative recovery.

The primary outcome variable was the composite score of the Quality of Recovery scale 15 at 24 and 48 h. Our hypothesis was that the active group should experience at least a 9-score difference, from the total score of 150, as compared to the control group of patients. A difference of 8 points has been assessed as clinically significant in a recent paper by the inventers of the scale [13]. Secondary outcomes was PONV and PDNV.

### 2.2 Statistics

Data is presented as mean plus minus standard deviation and frequencies as applicable. Differences in self-assessed quality of recovery was compared by Student-t-test. Differences between continuous data was compared with parametric tests; Student-t-test and category data was studied by Chi-square test. A p value less than 0.05 was considered statistically significant. Two groups of 36 patients each was required to show a difference with a p < 0.05 and a power of 80% based on a power analysis, a 9 score difference at 24-h between groups. We planned to include 76 patients to compensate for lost for follow-up. Data was analysed with StatView (v1.04) for MAC.

## 3 Results

**Fig. 1:**
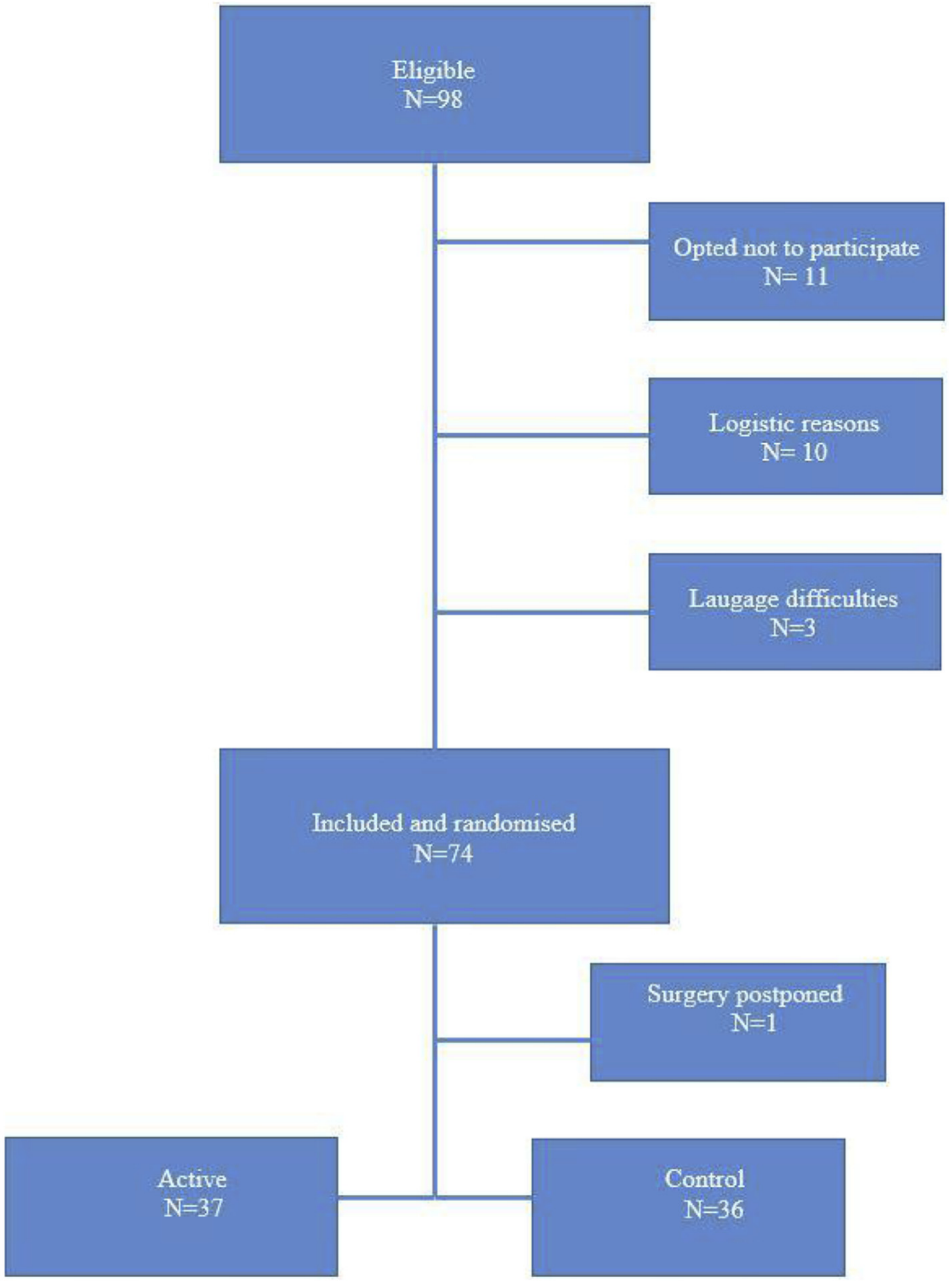
Consort.

Seventy-four ASA 1–2 female patients’ undergoing elective laparoscopic surgery were included, however surgery was postponed in one patient due to lack of surgery indication (See Fig. 1).

Seventy-three women, mean age 42 years (SD 12, 19–73), mean weight 72 kg (SD 10, 52–100), length 168 cm (SD 7, 155–186), BMI 26 (SD 3, 17–32) were included in the analysis. Demographics for the groups are presented in Table 2.

**Table 2:** Demographics.

	Active	Control
	n = 37	n = 36
Ase	43 ± 13	40 ± 12
Length	167 ± 7	168 ± 7
Weight	73 ± 10	71 ± 10
BMI	26 ± 3	25 ± 3
Smokers	5	3
Snuffers	2	1
Previous PONV	11	11
Mission sickness	17	16
Apfel score 2/3/4	4/23/10	1/25/10

Surgery and anaesthesia was uneventful. Perioperative observations are presented in a Table 3.

**Table 3:** Perioperative observations.

	Active n = 37	Control n = 36
Without food (mean ± SD)	12 ± 3	12 ± 4
Without fluids (mean ± SD)	4 ± 3	8*** ± 5
Ringer (mean ± SD)	670 ± 332	697 ± 248
Glucose (mean ± SD)	600 ± 234	667 ± 246
Duration anaesthesia (mean ± SD)	87 ± 26	94 ± 36
TIVA/inhalation (No. of Pat.)	33/4	35/1
Duration of surgery (mean ± SD)	57 ± 23	63 ± 27
Cholecystectomy/Gynaecological (mean ± SD)	32/5	29/7
Time in PACU (mean ± SD)	204 ± 112	232 ± 112
Apfel score 2/3/4 (mean ± SD)	4/23/10	1/25/10
Droperidol (mean ± SD)	24	25
Ondansetrone (mean ± SD)	15	19

***p < 0.001.

QoR15 mean value was significant higher in the active group at 24 h and 48 h. Results presented in the table QoR15 summary results.

Taking base-line preoperative scores into account 37 patients had not reached 90% of base value at 24 h (21 patients in the control group and 16 patients in the active group), 18 were still below 90% at 48 h’ score (9 controls and 9 active). There is no significant difference between the active and the control group in “recovered” however a small numeric difference see Table 5.

**Table 4:** QoR15 Summary results, mean ± SD.

	Active	Control
	n = 37	n = 36
Preoperative	120 ± 18	116 ± 19
2 h postoperative	115 ± 17	108 ± 21
24 h postop	113 ± 20	101 ± 25*
48 h postop	123 ± 13	111 ± 13***

*p < 0.05, ***p < 0.001.

**Table 5:** Recovered, number of patient reaching at least 90% of base-line sum QoR15 score.

24H	Active	Control
Unrecovered	16	21
Recovered	20	15
	N = 36	N = 36

48H	Active	Control
Unrecovered	9	9
Recovered	25	24
	N = 34	N = 33

## 4 Quality or recovery

### 4.1 Base-line, preoperative

There were differences in item 11 and 12 at base-line, moderate and severe pain; the control group scored worse for both mild and more intense pain; mean 7.7 SD 2.9 compared to 9.0 SD 1.9 for the active group (p = 0.033). For item 11, mild pain, control group mean 8.6 SD 2.8 vs 9.7 SD 1.3 for item 12 strong pain (p = 0.044). No further differences were noticed and sum score at base-line preoperatively is presented in table QoR15 summary results.

### 4.2 2-H postop

There were differences at 2 h in item 2 *enjoy food and drink* and item 4 *sleep quality* in favour for the active group of patients. Group mean for item 2 *enjoy food and drink*; control group scored lower mean 7.9 SD 3.1 compared to 9.3 SD 1.9 for the active group (p = 0.03) and item 4 *sleep quality*, control group lower mean 6.4 SD 3.3 compared to 8.1 in the active group (p = 0.011). The QoR15 sum did not differ between groups (see Table 4 QoR15 Summary results).

### 4.3 24-H postop

There were differences at 24 h in item 5 *hygiene and self-care,* item 13 *nausea and vomiting* and item 15 *depressed and anxious,* all in favour for the active group of patients. The control group scored lower on item 5 *hygiene and self-care* mean 8.5 SD 3.0 compared to 9.9 (SD 0.5) for the active group, item 13 *nausea and vomiting*, control group score mean 6.2 SD 3.9 compared to 8.3 SD 3.0 (p = 0.011) for the active group and item 15 *depressed and anxious* control group scored 8.5 SD 2.7 compared to 9.7 SD 1.0 (p = 0.02) for the active group.

The requested scores around intensity of nausea and dizziness were also lower in the active group at 24 h, mean nausea intensity score 0.6 SD 0.8 compared to 1.1 SD 1.2 in the control group (p = 0.021) and dizziness intensity score 0.5 SD 0.7 compared to 1 SD 0.7 (p = 0.003). There was also a difference in sum QoR15 scores see Table 4 QoR15 summary results.

### 4.4 48-H postop

There were item differences also at 48 h postop in mean group scores favouring active treatment in item 1 *easiness to breathe,* item 4 *sleep quality*, item 8 *capacity to work and take care of activities of daily living* and item 9 *control and comfort.* Item 1 *easiness to breathe* group mean score was higher in the active group 9.6 SD 1.1 compared to 8.8 SD 1.9 for the control group (p = 0.03). Item 4 *sleep quality* was better in active group at 48 h; mean 8.7 SD 2 compared to 7.5 SD 2.5 for the control group (p = 0.02). The item 8 *capacity to work and take care of activities of daily living* was better in the active group mean 7.7 SD 2.4 compared to 5.8 SD 3.7 for the control group (p = 0.02). Item 9 *control and comfort* was scored higher in active patients mean score 9.0 SD 1.4 compared to 7.9 SD 2.9 control group (p = 0.043). The mean sum QoR 15 score was significantly higher for the active compared to control group (see Table 4 QoR15 Summary results).

### 4.5 Nausea and vomiting

Nausea was experienced in 13 patients preoperatively. Postoperative nausea and vomiting was most commonly experienced during the first 24 h, 23 patients (12 controls and 11 active patients) experienced PONV in the recovery area. In all, 39 patients (53%) reported PONV/PDNV on the 24-h questionnaire (22 control patients and 17 active). Day 2, 22 patients still experienced nausea but no one had vomited (12 control patients and 10 active).

**Fig. 2:**
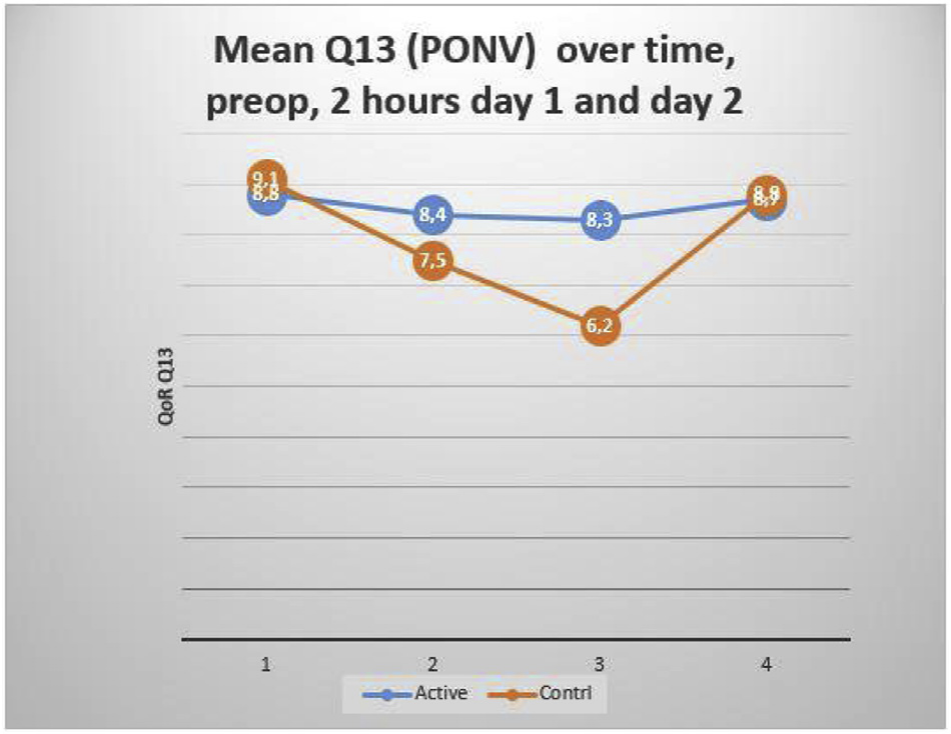
Mean Q13 over time, preop, 2 h day 1 and day 2.

Over all 50 patients (68%) had experienced PONV/PDNV during the 48-h follow-up period, 25 in each group. The day 1 patient's assessed PONV intensity showed lower numeric intensity among active patients' median 1 range 1–7 vs. median 2 range 0–8 among control females and the mean QoR score for nausea was lower at 24-h for the active group see Fig. 2. Nausea and vomiting significantly reduced the QoR score at all 3 time-points assessed.

## 5 Discussion

The aim of this study was to assess whether possible to improve patients’ self-assessed quality or recovery by simply provide female patient undergoing laparoscopy surgery with a preoperative 200 ml nutritional drink and chewing gum during early recovery. At 24 h and 48 h we could show a better quality of recovery mean score for women in the active group and it was clear that PONV/PDNV had a negative impact on the quality of recovery. We have chosen to report certain items individually to try to show were the differences between the groups were.

There is sparse data around multi-dimensional preoperative assessment of patients scheduled for general anaesthesia. Preoperative anxiety was found to be significant and with impact on quality of recovery in a study by Sadati et al. assessing the effects of preoperative nursing intervention to reduce preoperative fear [16]. Ay et al. found likewise that low level of education, being of the female sex, being single, and having laparoscopic operation were factors related to preoperative anxiety [17]. It should be acknowledged that our patients did not receive any anxiolytic premedication. Preoperative information and preparation were standardised, in accordance to department routines for both groups.

Recovery following surgery takes time. Royse et al. studied quality of recovery using the Postop Quality of Recovery scale. They found recovery, back to base-line scores was a long-lasting process over weeks also for minor procedure, knee arthroscopy, tonsillectomy and nasal surgery [18,19]. Le et all found that QoR40 scores were back at base-line day 2 in a study comparing TIVA and desflurane in female patients undergoing thyroid surgery [20]. We found in a previous study that cognitive recovery assess by the Postoperative Quality of Recovery scale cognitive test was still incomplete for twenty percent of patients at 48 h and with no significant difference between female breast cancer patients having had desflurane and TIVA based anaesthesia [21]. Difference in QoR15 sum score was not seen in the day of surgery. A difference between the two groups was seen at 24 h and there was a difference, benefit for the active treatment, at 48 h. Twenty-four percent of patients had still not reach 90% of base-line which raise the question how long the recovery time to base-line are for those women. A more protected follow-up would have been of interest. Still our results are not in line with Surender et al. They used the Quality of recovery scale 40 for assessment quality of recovery comparing 0.1 mg/kg dexamethasone to 0.2 mg/kg lidocaine intravenously and in 67 females undergoing cholecystectomy with inhalational (sevoflurane) anaesthesia and found a mean score of 188 at preoperative base-line and return of score already at 24 h [22]. Lee JS et al. used also the Quality of recovery scale 40 for assessment of recovery at 24 h comparing nutritional drink in patients undergoing laparoscopic cholecystectomy in general anaesthesia based on inhalation and remifentanil [23]. They found that the recovery score was back to base-line already at 24-h, but no difference between the patients having preoperative nutritional drink. We cannot explain the difference in results, however one should acknowledge that we assessed combined the nutritional drink with chewing gum, with possibly additive effects. One can merely speculate whether Swedish females are more critical around the expectations of recovery.

We had hoped for an impact not only on overall quality of recovery but also on the experience on PONV/PDNV. The effects of our intervention, nutritional drink and chewing gum was however very minor, possibly providing a less intense PONV experience the first 24 h. One could speculate to the fact that the chewing gum should have impact primary to early PONV e.g. in the first hours of recovery there we did not see a difference between the two groups and therefore be hesitant to the efficacy of the intervention. It should be acknowledged that PONV prophylaxis was administered by attending anaesthetist. It was expected that the PONV prevention routines were to be followed, but deviations in risk score-based prevention was noticed.

### 5.1 Limitations

The study was not blinded, the bias from the intervention per see must indeed be acknowledged. Our results must also be put into the context of some differences between the groups. There was significant difference between the active and control groups fasting from fluids time. Overall our patients were withholding fluid longer than set guidelines. This raise the question of the importance to preoperative guidelines of fluid intake. Maybe our information to females planned for abdominal surgery always should point out the benefits of fluid intake up to 2 h prior to surgery as according to the ERAS program, and with this avoid long fasting periods. The control group showed also a worse base-line in several of the QoR items. Another limitation in this study is the fact that PONV prophylaxis were given to according to the anaesthetist in charge, which not always were according to current guidelines. Although this was the case for both control and active group and there was no significant difference between the two groups.

## 6 Conclusion

Recovery after laparoscopic surgery takes time. The benefit vs. risk for our intervention seems positive; the simple intervention providing a preoperative nutritional drink and letting the patient have a chewing gum during recovery was found to have benefits on quality of recovery. The negative impact of PDNV should be recalled and further studies aiming at improving quality of recovery following laparoscopic surgery in females are warranted.

## Provenance and peer review

Not commissioned, externally peer reviewed.

## Ethical approval

Ethics Committee at the Karolinska Institute (Dnr: 2016/2107–31/4).

## Sources of funding

Department funding only

## Author contribution

All authors have been taking active part in the project.

## Conflicts of interest

None.

## Research registration number

The study is registered in ClinicalTrails.gov, NCT03825666.

## Guarantor

Emma Öbrink.
